# Repurpose and Reuse: Artistic Perspectives on Antimicrobial Resistance

**DOI:** 10.3201/eid2501.AC2501

**Published:** 2019-01

**Authors:** Byron Breedlove

**Affiliations:** Centers for Disease Control and Prevention, Atlanta, Georgia, USA

**Keywords:** art science connection, emerging infectious diseases, Repurpose and reuse: artistic perspectives on antimicrobial resistance, Make do and mend, Anna Dumitriu, antimicrobial resistance, art and medicine, Alexander Fleming, penicillin, CRISPR, bioart, about the cover

**Figure:**
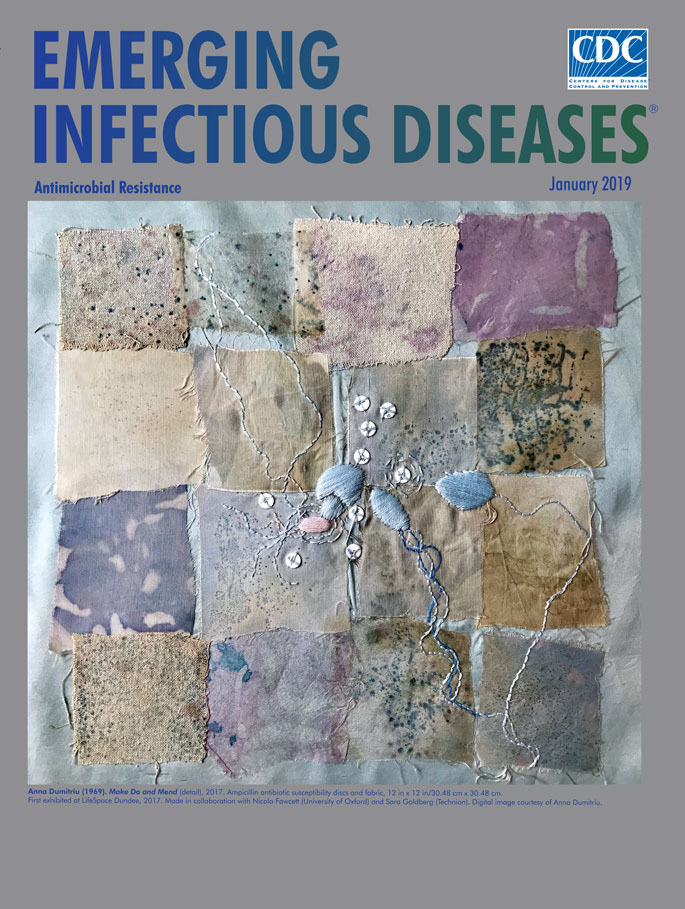
**Anna Dumitriu (1969). “Make Do and Mend” detail, 2017**. Ampicillin antibiotic susceptibility discs and fabric, 12 in × 12 in/30.48 cm × 30.48 cm. First exhibited at LifeSpace Dundee, 2017. Made in collaboration with Nicola Fawcett (University of Oxford) and Sarah Goldberg (Technion). Digital image courtesy of Anna Dumitriu.

Antimicrobial resistance ranks among the most urgent global challenges of the 21st century. When penicillin became widely available in 1943, the specter of antimicrobial resistance was already stalking this seemingly miraculous drug. As new antimicrobial agents have been developed, they, too, have gradually lost effectiveness because of their misuse and overuse for human, animal, and agricultural health.

British artist Anna Dumitriu, who served as the 2018 president of the Science and the Arts Section of the British Science Association, has focused on the issue of antimicrobial resistance in her recent work. She states, “We are confronted by a very difficult situation now, where important antibiotic drugs we have relied on for many years have simply stopped working, because bacteria have evolved strategies to beat them. I’m fascinated how the drug discovery process works, how infectious diseases were treated in the past, and in what is happening now in scientific research to improve health.”

Working with an array of traditional fine arts and craft materials and with bacteria, antibiotics, and DNA sequences, Dumitriu melds microbiology with fine art to create works within the genre of “bioart.” Interestingly, Sir Alexander Fleming, discoverer of penicillin, created some of the earliest examples of this art form. He indulged his creative side by using laboratory instruments to “paint” ephemeral figures and landscapes, growing microbes with different natural pigmentations on agar-filled petri dishes and waiting for the images to develop.

Dumitriu is among the vanguard of a small cadre of interdisciplinary practitioners who wield their creative skills in studios and laboratories. Featured on this month’s cover art is one component of her 2017 project *Make Do and Mend*, a section of quilt that comprises 16 irregular silk squares. The brown, blue, pink, and plum squares are flecked and dappled with splotches of contrasting colors, and assembled into a quilt with a combination of backstitch, running stitch, and satin stitch. As is the case with many quilts, the final product came from a cooperative effort—in this case, the artist and her scientific collaborator Nicola Fawcett.

Each silk square is stained with diluted fecal samples from individual patients in Oxford, UK, who had consented to their samples being used in artworks. Those samples were grown on silk cloth squares using chromogenic agar. The blue/pink patches that display different-sized colonies of bacteria indicate a diverse gut microbiome. All-blue or all-pink sections suggest a gut microbiome that has reduced diversity from antibiotic use. Clustered near the center are three embroidered shapes with trailing flagella that represent *Escherichia coli* bacteria. Nine ampicillin susceptibility discs are stitched into the quilt. (For anyone interested in protocols, Fawcett states, “Dumitriu works with expert microbiologists to integrate compliance with health and safety standards into her work.”)

In another aspect of this project, Dumitriu and Dr. Sarah Goldberg used CRISPR (short for clusters of regularly interspaced short palindromic repeats) to edit the genome of a strain of *E. coli* bacteria. They removed an ampicillin antibiotic resistance gene and replaced it with a fragment of DNA (converted into ASCII code and then to base 4) that encoded the World War II slogan “Make Do and Mend”. That slogan came from the title of a pamphlet issued by the British Ministry of Information encouraging homemakers to repair and reuse clothing during wartime. The cover of that leaflet is also part of the exhibit.

Dumitriu conceived of this exhibit as a way to commemorate the 75th anniversary of the first use of penicillin in patients and to increase awareness about the rapid development of antibiotic-resistant strains of pathogens. Her *Make Do and Mend* project may help stimulate creative thinking about antimicrobial resistance and stewardship. Perhaps new serendipitous breakthroughs will allow us to repurpose and reuse some of our diminished antimicrobials in keeping with Fleming’s famous quote, “One sometimes finds what one is not looking for.”
